# Evaluation and Expression of Survivin in Potentially Malignant Lesions and Squamous Cell Carcinoma: A Comparative Study

**DOI:** 10.7759/cureus.7551

**Published:** 2020-04-05

**Authors:** Rekhaa Sakthivel, Ananthalakshmi Ramamoorthy, Nadeem Jeddy, Mamta Singaram

**Affiliations:** 1 Oral Pathology, Thai Moogambigai Dental College and Hospital, Chennai, IND; 2 Oral and Maxillofacial Pathology, Thai Moogambigai Dental College and Hospital, Chennai, IND; 3 Oral and Maxillofacial Surgery, Thai Moogambigai Dental College and Hospital, Chennai, IND

**Keywords:** survivin., premalignant lesions, squamous cell carcinoma, potentially malignant lesions, oral squamous cell carcinoma

## Abstract

Introduction

Overexpression of survivin, an anti-apoptotic protein, has been associated with the progression of cancer, resistance to drugs, and a poor prognosis. The expression level of survivin indicates the progression of the disease, early recurrence, and a failure to respond to therapy. Our study was a retrospective analysis performed on archival specimens.

Materials and methods

The study included a total of 50 histopathologically proven cases of potentially malignant lesions and squamous cell carcinoma. Immunohistochemical staining was carried out using primary rabbit monoclonal antibodies to survivin (PathnSitu, Telangana, India) along with a horseradish peroxidase detection kit (Leica Biosystems, Maharashtra, India). The intensity of staining of survivin in the epithelium was determined, and the data obtained from potentially malignant lesions, oral squamous cell carcinoma, fetal tissue, and normal oral mucosa were compared.

Results

The expression of survivin was positive in 70% of the samples of oral squamous cell carcinoma followed by 50% from cases of leukoplakia, 20% of oral submucous fibrosis samples, and 10% of lichen planus samples (P < 0.05).

Conclusion

Malignant transformation of these potentially malignant lesions increases with increased expression of survivin. This expression of the anti-apoptotic protein might be an early phenomenon in the initiation and advancement of oral squamous cell carcinoma. The prognosis of oral squamous cell carcinomas becomes poorer with increased expression of survivin. Therefore, survivin might be helpful as an important therapeutic target because it is expressed more in tumor cells and absent in most adult tissues.

## Introduction

Survivin is an anti-apoptotic protein encoded by the BIRC5 gene (baculoviral inhibitor of apoptosis [IAP] repeat-containing protein 5) in humans. Survivin, a member of the IAP family, is unlike other IAP proteins and is found during embryonic and fetal developmental stages [1]. However, survivin is down-regulated and almost undetectable in healthy adult tissues. However, it becomes expressed in most common human cancers; survivin is completely absent in terminally differentiated cells and is expressed highly in most human tumors and fetal tissue [2]. These data suggest that survivin might provide a new target for cancer therapy that would discriminate between healthy and transformed cells. The overexpression of survivin has been correlated with cancer progression, drug resistance, and poor prognosis [3]. Because of its expression in malignancy, it is of great interest as both a tumor diagnostic and a prognostic marker, and also as a new biologic target for future anticancer therapies. The expression rate of survivin is indicative of disease progression, early recurrences, and resistance to therapy [4]. This study aims to estimate the role of survivin in the carcinogenesis of premalignant to malignant tumors and its prognostic role in oral squamous cell carcinoma (SCC).

## Materials and methods

This was a retrospective study performed on archival specimens. The study included a total of 50 histopathologically proven cases of leukoplakia, oral SCC, lichen planus, oral submucous fibrosis, normal oral mucosa samples, and fetal tissue. Paraffin-embedded blocks of all the samples were obtained from the archives of the Department of Oral Pathology and Microbiology, Thai Moogambigai Dental College and Hospital, Chennai, India. Hematoxylin and eosin staining were performed to confirm the diagnosis. Immunohistochemical staining was carried out using primary rabbit monoclonal antibodies to survivin (PathnSitu, Telangana, India) along with a horseradish peroxidase detection kit (Leica Biosystems, Maharashtra, India). Four-micrometer sections of formalin-fixed and paraffin-embedded tissues were used for the immunohistochemistry study and were mounted on (3-aminoproypl)triethoxysilane-coated slides. The intensity of survivin staining in the epithelium was ascertained. Data obtained from potentially malignant lesions, oral SCC, fetal tissue, and normal oral mucosa were analyzed.

## Results

A summary of the results is shown in Table 1. There was a pronounced expression of survivin in oral SCC followed by lower levels in oral potentially malignant lesions. The expression of survivin was positive in 70% of oral SCC samples followed by 50% of leukoplakia samples, 20% of oral submucous fibrosis samples, and 10% of lichen planus samples (P < 0.05). All samples of normal oral mucosa were negative for the expression of survivin. Among the fetal tissue group, all samples were positive.

**Table 1:** Expression of survivin in oral submucous fibrosis, lichen planus, leukoplakia, oral squamous cell carcinoma, normal oral mucosa, and fetal tissue

Groups	Survivin		P-value
	Positive	Negative	
Oral submucous fibrosis	3	7	0.034
	30%	70%	
Lichen planus	2	8	
	20%	80%	
Leukoplakia	5	5	
	50%	50%	
Squamous cell carcinoma	7	3	
	70%	30%	
Normal	0	5	
	0.0%	100%	
Fetal	5	0	
	100%	0%	
Total	22	28	

## Discussion

Oral carcinoma is the most common cancer of the head and neck region. The five-year survival rate for oral SCC is only 35% to 50% despite the advanced treatment therapy and the emergence of aggressive chemotherapy regimens [5]. One of the major causes of the unfavorable prognosis of cancer of the oral cavity is the inability to detect the disease at an early stage and its subsequent progression. Therefore, it is necessary to find a specific molecular tumor marker for predicting tumor progression and disease prognosis.

A tumor develops when there is an imbalance in the regulation of cell proliferation and control of cell death. Survivin, a potential tumor marker, is known to be responsible for tumor cell viability, resistance to apoptosis, and magnification of tumor progression [6]. Survivin is involved in the inhibition of terminal effector caspase-3 and caspase-7 and eventually prevents apoptosis. Survivin is always expressed in fetal cells but not in differentiated adult tissue. Survivin, an IAP group member, also plays a role in the development and progression of carcinoma, resulting in the expression of survivin [7]. Therefore, survivin expression can help evaluate the progress of head and neck carcinomas.

In this immunohistochemical study, survivin was detected in potentially malignant oral disorders and SCC; its expression was evaluated in each group.

In the fetal tissue group, all the samples in our study were positive. Survivin is strongly expressed in embryonic and fetal organs but had not been reported in differentiated healthy tissues; we confirmed these results in our study [8]. Several studies indicate that survivin can be detected in the embryonic stages. Survivin was expressed during embryonic and fetal developmental stages, suggesting its different roles in these stages in a study conducted by Bowen et al. [9]. Survivin is a member of the IAP protein family found during embryonic and fetal developmental stages [10].

The healthy mucosa group was negative for survivin in all samples; this was in agreement with the previous observations made by Lo Muzio et al. in 2003 [11]. In his study, the healthy oral mucosa specimens were positive only in sporadic cells of the basal and parabasal layers and were considered negative for survivin expression by immunohistochemistry. While several studies demonstrated survivin expression in basal and parabasal cells of the oral mucosa, studies employing immunochemistry techniques reported no survivin expression in clinically normal oral mucosa.

The survivin expression level in the lichen planus group was 20%, suggesting a role for this marker in the early stages of carcinogenesis. A lack of its expression observed in other samples of lichen planus might be due to the nature of the potentially malignant lesion. The findings demonstrated in this study ate in agreement with another immunohistochemical study performed by Lo Muzio et al. in 2005 that investigated the potential predictive value of survivin as an early predictor of malignant transformation in precancerous lesions and oral SCC [12]. The results of this study ate also in agreement with a 2009 immunohistochemical study conducted by Oluwadara et al., confirming the expression of survivin in patients with oral lichen planus [13]. Survivin levels in normal oral mucosa, oral leukoplakia, and oral SCC are measured using reverse transcription-polymerase chain reaction [14]. All samples of normal oral mucosa showed very low/nearly undetectable survivin levels, whereas the highest survivin expression values were seen in the cancer group, which is comparable to our study.

Among 10 oral submucous fibrosis cases, 30% were positive (three samples) and 70% were negative, again suggesting the role of survivin in carcinogenesis. The oral submucous fibrosis samples were not graded during the study, and hence advanced stages of oral submucous fibrosis cases would have been expected to show more expression of survivin. Previous literature shows very little work on survivin expression in oral submucous fibrosis; hence, our results could not be compared. Since the malignant transformation of oral submucous fibrosis is low (3% to 7.6%), survivin expression may be poor in this study [14].

Of the leukoplakia samples, 50% were positive for survivin. Thus, we postulate that during the development of oral cancer, survivin might accumulate within the involved tissue at an early stage. These results were similar to the study conducted by Negi et al. in 2015, indicating that a high incidence of survivin protein expression in oral epithelial dysplasia may be an early event in carcinogenesis [5]. The putative predictive value of survivin was also reported by Lo Muzio et al, where a higher rate of positive samples among lesions evolving into SCC compared with lesions not evolving was shown [15]. Also, they found no signiﬁcant correlation between survivin expression and the degree of dysplasia. One limitation of our study is the small number of samples. Larger groups with dysplastic leukoplakia would have allowed a more robust analysis of the relationship between a well-known risk factor of malignant transformation (dysplasia) and a putative one (survivin expression).

In the SCC group, survivin expression was positive in 7 of 10 samples, confirming the role of this protein in the carcinogenesis process. The histopathological grading of SCC as well-differentiated, moderately differentiated, and poorly differentiated was not included in our study and hence could not be compared with other studies. A 2003 study by Lo Muzio et al. inferred that survivin is expressed in more than 80% of the cases of oral SCC [15]. In particular, survivin expression was often increased in poorly differentiated tumors, and it was concluded that survivin serves as a potential novel molecular marker of aggressive neoplasia. A previous study in 2000 by Chen et al. demonstrated that the expression of survivin in oral SCC could potentially contribute to tumor progression [16]. This connection to progression was suggested by the increased expression of survivin in poorly differentiated oral SCCs as compared with histological normal oral epithelium.

A study by Li et al. inferred that the interference with survivin function induces pleiotropic cell division defects and apoptosis, suggesting a potential role in the interface between cell division and apoptosis control [17]. In a study by Chiodino et al., the presence of survivin in more aggressive and poorly differentiated variants of oral SCC confirmed and extended earlier reports of survivin expression in cancers and suggested its potential predictive and prognostic impact for disease progression [18].

The non-fetal expression of survivin in cancer specimens and potentially malignant lesions correlates the role of this protein in carcinogenesis. Since only a histopathological diagnosis was taken into consideration and not the clinical data, future studies should include the age of the patient, habits, and site of the lesion, and a different technique should be carried out for more significant results. Also, survivin expression is known to be reduced in virus-induced oral SCC and increased in tobacco-induced oral SCC, as shown by Lodi et al. [19]. Since the previous literature indicates that survivin expression differs with tobacco consumption, viral etiology, and the site of involvement in the oral cavity, and since our study did not involve any of the aforementioned factors, this is a limitation of our study that should be rectified with a larger sample size so that more light could be shed on survivin. Finally, as our study is an evaluative one and not a quantitative study of survivin expression, the interpretation may differ according to the observer.

## Conclusions

This study aimed to evaluate and compare the immunohistochemical expression of survivin in potentially malignant lesions and with that of oral SCC. The expression of survivin was predominantly seen in the oral SCC samples followed by premalignant lesions, and this was statistically significant. The up-regulation of survivin in potentially malignant lesions and oral SCC provides evidence for its role in carcinogenesis. The pronounced expression of survivin may potentially be used as a reliable marker to identify the high-risk group for malignant transformation. Since the malignant transformation of the potentially malignant lesions correlates with increased expression of survivin, and the prognosis of oral SCCs becomes poorer with increased expression of survivin, future studies should include histopathological grading of SCCs and potentially malignant lesions for more interesting results. With the intent of reducing the mortality and morbidity of oral SCC, survivin may become a target for anticancer strategy in conjunction with the existing treatment modalities.
